# Genome-Wide Associations of Gene Expression Variation in Humans

**DOI:** 10.1371/journal.pgen.0010078

**Published:** 2005-12-16

**Authors:** Barbara E Stranger, Matthew S Forrest, Andrew G Clark, Mark J Minichiello, Samuel Deutsch, Robert Lyle, Sarah Hunt, Brenda Kahl, Stylianos E Antonarakis, Simon Tavaré, Panagiotis Deloukas, Emmanouil T Dermitzakis

**Affiliations:** 1 Wellcome Trust Sanger Institute, Wellcome Trust Genome Campus, Hinxton, United Kingdom; 2 Department of Molecular Biology and Genetics, Cornell University, Ithaca, New York, United States of America; 3 Department of Genetic Medicine and Development, University of Geneva Medical School, Geneva, Switzerland; 4 Illumina, Inc., San Diego, California, United States of America; 5 Department of Oncology, University of Cambridge, Hutchison/MRC Research Centre, Cambridge, United Kingdom; 6 Program in Molecular and Computational Biology, University of Southern California, Los Angeles, California, United States of America; University of Alabama at Birmingham, United States of America

## Abstract

The exploration of quantitative variation in human populations has become one of the major priorities for medical genetics. The successful identification of variants that contribute to complex traits is highly dependent on reliable assays and genetic maps. We have performed a genome-wide quantitative trait analysis of 630 genes in 60 unrelated Utah residents with ancestry from Northern and Western Europe using the publicly available phase I data of the International HapMap project. The genes are located in regions of the human genome with elevated functional annotation and disease interest including the ENCODE regions spanning 1% of the genome, Chromosome 21 and Chromosome 20q12–13.2. We apply three different methods of multiple test correction, including Bonferroni, false discovery rate, and permutations. For the 374 expressed genes, we find many regions with statistically significant association of single nucleotide polymorphisms (SNPs) with expression variation in lymphoblastoid cell lines after correcting for multiple tests. Based on our analyses, the signal proximal *(cis-)* to the genes of interest is more abundant and more stable than distal and *trans* across statistical methodologies. Our results suggest that regulatory polymorphism is widespread in the human genome and show that the 5-kb (phase I) HapMap has sufficient density to enable linkage disequilibrium mapping in humans. Such studies will significantly enhance our ability to annotate the non-coding part of the genome and interpret functional variation. In addition, we demonstrate that the HapMap cell lines themselves may serve as a useful resource for quantitative measurements at the cellular level.

## Synopsis

With the finished reference sequence of the human genome now available, focus has shifted towards trying to identify all of the functional elements within the sequence. Although quite a lot of progress has been made towards identifying some classes of genomic elements, in particular protein-coding sequences, the characterization of regulatory elements remains a challenge. The authors describe the genetic mapping of regions of the genome that have functional effects on quantitative levels of gene expression. Gene expression of 630 genes was measured in cell lines derived from 60 unrelated human individuals, the same Utah residents of Northern and Western European ancestry that have been genetically well-characterized by The International HapMap Project. This paper reports significant variation among individuals with respect to levels of gene expression, and demonstrates that this quantitative trait has a genetic basis. For some genes, the genetic signal was localized to specific locations in the human genome sequence; in most cases the genomic region associated with expression variation was physically close to the gene whose expression it regulated. The authors demonstrate the feasibility of performing whole-genome association scans to map quantitative traits, and highlight statistical issues that are increasingly important for whole-genome disease mapping studies.

## Introduction

Mapping genetic factors that underlie quantitative traits in humans has been a challenging task in the absence of large samples with accurate phenotypic measures and dense genotypic data. In particular, one of the gaps in our knowledge of human biology is the structure of genetic variation affecting gene regulation and how it contributes to phenotypic variation and disease [1]. Recent studies in model organisms including yeast [2–5], mouse [6–8], maize [8], and rat [9], have attempted to address this issue by testing for linkage and/or associations of gene expression variation among individuals with nucleotide variation. As a result, extensive functional genetic variation has been discovered, suggesting that the overall contribution of regulatory variation to phenotypic variation has been underestimated. In humans, three studies have followed a two-stage approach: first performing linkage analysis to identify regions in which gene expression variation segregates in pedigrees, and then linkage disequilibrium (LD) mapping those regions in a larger sample of unrelated individuals with additional markers [10–12]. Linkage analysis may miss weaker signals in the first stage, as it relies on sufficient differences in phenotypic means among recombinant and non-recombinant genotypes [13]. On the other hand, allele-specific expression experiments can identify signals nearby the gene and in LD with the coding single nucleotide polymorphism (SNP) that is used for the measurement, but cannot provide an unbiased view of regulatory variation in the human genome [14,15]. Association studies have generally more power to detect such signals [16], and the availability of high throughput methods for genotyping and gene expression profiling make genome-wide scans an appealing alternative. But genome-wide studies pose their own challenge in the form of statistical inference in the face of so many simultaneous statistical tests. Obtaining sufficient power using a test with a given rate of false positives in this setting remains a serious challenge [17].

In this study we employed the densely genotyped (>1 million SNPs) HapMap panel of 60 unrelated US residents of Northern and Western European descent (labeled CEU; see [18,19]) to perform a genome-wide association study of gene expression in 630 genes (excluding control genes). We show that even with this limited sample size, we are able to detect strong and highly significant SNP-to-expression associations, most of which are *cis* (proximal 1-Mb regions) to the gene of interest. This analysis provides the first unbiased and genome-wide view of regulatory associations in samples of unrelated individuals in humans.

## Results

### Sample and Experimental Design

We selected a set of 630 protein coding genes (see Figure S1 for chromosomal distribution) consisting at the time (January 2004) of all Refseq genes in the ENCODE regions [20] (321 genes, excluding the HSA21 ENCODE regions), all Refseq genes on human Chromosome 21 (191 genes), and all Refseq and manually curated genes from a 10-Mb region at 20q12–13.2 (118 genes), and surveyed their transcript levels in a sample of lymphoblastoid cell lines generated from 60 unrelated CEU individuals. Transcript levels were measured with the Illumina BeadArray technology using two or more unique oligonucleotide probes per gene (1,433 probes including controls; see Materials and Methods) [21]. We performed a minimum of six replicate hybridizations for each of the 60 samples and obtained expression values for all the probes and samples. We normalized the raw intensity values with a quantile normalization method and averaged the replicate values (see Materials and Methods) per probe per sample to obtain a single expression level per individual per probe. We found excellent correlation between measurements of replicates within and between arrays (r^2^ = 0.96–0.99).

### Initial Association Analysis

To perform association tests between gene expression variation and SNP variation, we selected 374 of the 630 tested genes that had probe hybridization signals significantly above the background and were among the most variable (we excluded probes with phenotypic outliers; see Materials and Methods), corresponding to 688 unique probes, and the public release of 753,712 SNPs with minor allele frequency above 5% made by the HapMap project (HapMap version 16b; [19]). The actual determinants of gene expression levels are likely to be associated with many interacting factors across the genome, but in light of the small sample size, we consider here only the simplest models testing for single-SNP effects. We employed a main effects, linear regression model separately for each probe, where the additive effect of a SNP genotype is tested by coding the genotypes of each SNP as 0, 1, and 2 (corresponding to the counts of the minor allele in each genotype) and performing a linear regression of the new variable, “allele counts”, with the normalized gene expression values; this test has one degree of freedom. Note that we treated each probe separately to account for potential differences between the transcript levels inferred by the one or the other due to SNPs or other sequence dependent effects, though in most cases the two probes of each gene were highly correlated (see Figure S2) and produced almost identical results. This makes it unlikely that the associations described below are artifacts of segregation of SNPs within the 50-mer probe sequence but we describe a more detailed analysis below to account for such effects. Because the model considers the effects of each SNP singly, it cannot test the role of potential SNP–SNP interaction effects.

### Methods for Multiple-Test Correction

One of the main problems of performing genome-wide analysis with such a large number of phenotypes and SNPs is correcting for multiple testing. Despite the fact that there have been a number of approaches to handle this problem, it is not yet clear which is the optimal way to perform such correction [22]. Thus we applied three methodologies routinely employed for multiple test correction when using a random set of markers from the genome, namely the Bonferroni correction for multiple tests, generating a null distribution of *p*-values by permuting the phenotypes relative to the genotypes [23], and the false discovery rate (FDR) [24]. In all cases of multiple correction we assigned a significance threshold of *p* = 0.05 after the correction.

**Figure 1:**
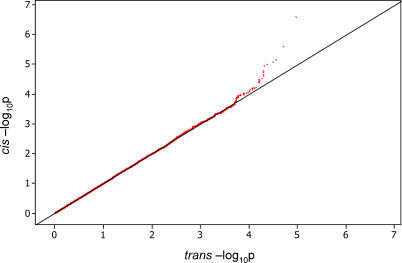
QQ Plot of *cis* versus *trans* HSA20 −log_10_ *p*-Values The figure shows the contrast of −log_10_ *p*-values deriving from associations of SNPs and genes within the 10-Mb region of HSA20 with −log_10_ *p*-values deriving from associations between genes on the 10-Mb region HSA20 with SNPs in one of ten ENCODE regions. Note that the distribution falls off the diagonal around −log_10_ *p* = 4, which we consider the borderline for the high enrichment of *cis* significant effects. A similar pattern is observed with any set of *trans* −log_10_ *p*-values on HSA20 or any other *cis* vs. *trans* contrast in any region we tested.

We considered both the genome-wide distribution of *p*-values as well as a subset of SNPs within 1 Mb from the genes tested. The rationale for the 1-Mb subset is that most of the *cis* regulatory regions of a gene are located within a small distance from the gene (though known enhancers can be as far as 1 Mb away from the gene; [25]) and therefore the density of relevant sequences around each gene is high. This can be viewed as a “candidate region” approach similar to the candidate gene approach used in disease studies. By restricting our analysis to these candidate regions we are more likely to detect relevant associations. This was evident when we contrasted the distribution of *p-*values between *cis* and *trans* (Figure 1). For the purposes of our analysis we have defined *cis* to be 1 Mb from the midpoint of the genomic region of the corresponding gene.

Bonferroni correction was applied to the genome-wide analysis as well as to a subset of the associations where SNP−gene distance was less than 1 Mb. The *p*-values generated from the individual SNP−gene tests were evaluated according to an adjusted significance threshold generated by dividing the 0.05 threshold by the total number of tests (number of genes multiplied by number of SNPs) performed in each case (whole genome or *cis*). The purpose of applying the Bonferroni correction to the 1-Mb subset of SNPs was to assess significance when only the *cis-* signal is considered. The whole genome Bonferroni correction was based on all 753,712 SNPs. If we had performed a whole genome analysis using a subset of “tag” SNPs, for example using 500,000 SNPs, the Bonferroni significance threshold would have differed little (*p*-value equal to 2.67 × 10^−10^ instead of 1.77 × 10^−10^; corresponding to −log_10_ *p* equal to 9.57 and 9.75, respectively).

In order to obtain significance values using an empirical null distribution of *p*-values from the observed data, we performed 12,500 permutations, wherein each permutation shuffled the expression values relative to the genotypes and for each permutation we retained the minimum *p*-value for each gene separately [26]. Empirical *p*-values were obtained by comparing the observed (non-permuted) *p*-values for each gene to the distribution of permuted *p*-values for the same gene. We subsequently corrected for the number of genes tested by adjusting the significance threshold by dividing by the number of genes tested.

The permutation analysis was first performed using genotypes for all 753,712 SNPs, and then again using genotypes for only those SNPs within 1 Mb of the gene of interest to test the significance of *cis* effects. The permutations were performed separately for each of the two sets of SNPs according to the description above, and significance was assessed for each using the same implementation of multiple test correction as described.

For the third test, we employed an FDR analysis [24], considering only those *p*-values from associations of all tests with SNP−gene distance less than 1 Mb. The distribution of all of these *p*-values together (i.e., *p*-values from all genes and their *cis* SNPs) was used to calculate the FDR and to assess significance of each individual *p*-value in the distribution. A whole-genome FDR calculation was not undertaken as the number of genome-wide *p*-values made it computationally prohibitive to run the analysis. Signals were considered significant if a *p*-value had a corresponding *q-*value of less than 0.05.

### Assigning Significance to Associations

**Figure 2:**
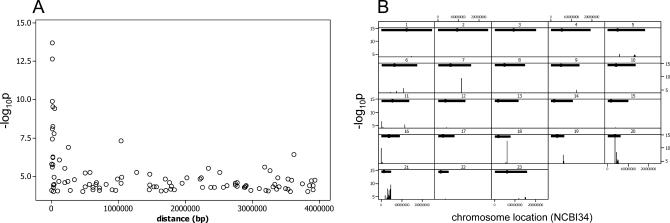
*Cis-* Signals of SNP−Gene Associations in the Human Genome (A) The relationship between statistical significance and distance from gene. Each data point represents the maximum −log_10_ *p* for a single gene and SNPs located *cis-* to its coding locus. The −log_10_ *p*-values from the additive model are plotted as a function of distance between the center of the genomic span of the gene and *cis-* located SNPs (*cis-* < 4 Mb). Only those gene-SNP associations that have −log_10_ *p* > 4 are shown. SNPs are from the 5-kb HapMap. This plot includes data for 101 genes (129 probes). (B) *Cis-* SNPs with −log_10_ *p* ≥ 4 from the 688 probes analyzed are plotted against their chromosomal location on NCBI34 coordinates of the human genome.

We detected highly significant (−log_10_ *p* up to 13.6) and multiple associations between gene expression level and SNPs in close proximity (less than 400 kb) to the corresponding gene. The signal from the additive model decayed rapidly with distance from the gene (Figure 2A and 2B). In particular, based on the Bonferroni genome-wide threshold *(Bonf-gw)* we detected a total of 99 SNPs (183 SNP-probe associations) that significantly explained transcript levels of six genes, half of which (three out of six) are in *cis* and half (three out of six) are in *trans*. These 99 SNPs cluster into seven genomic regions. When we applied the Bonferroni correction only for SNP−gene associations with distances less than 1 Mb *(Bonf-cis)* the number increased to 171 SNPs (310 SNP-probe associations) for ten genes, with these SNPs clustering into ten genomic regions.

Permutation-based assignment of significance was also applied to our analysis. When we performed genome-wide correction *(Perm-gw)* only three genes had significant associations, and these were in *cis*. The same three *cis* associations were identified by the genome-wide Bonferroni correction. Interestingly, no *trans* associations were significant with the permutation-based analysis; notably absent were the three *trans* associations identified by the genome-wide Bonferroni correction. This suggests that although Bonferroni is generally a conservative correction method, it might still identify spurious associations if the assumptions of the original parametric model are violated (see below). Permutation-based correction of *p*-values for SNP−gene distances of less than 1 Mb *(Perm-cis)* detected ten genes with significant associations, which were the same ten genes identified by the 1-Mb *Bonf-cis* correction. To further explore the signal *cis* to the genes tested, and uncover associations that might be biologically relevant but do not cross the significance threshold due to our small sample size, we considered all the genes that were individually significant at the 0.05 significance threshold based on the permutation test *(Perm-cis-enrich).* A total of 63 genes were determined to be individually significant at this level, while we expect only 19 by chance. This pool of 63 genes is therefore enriched by 44 genes that appear to have significant signals within 1 Mb of the gene.

Finally, we assigned significance based on a FDR of *q* = 0.05. As mentioned above, it was computationally prohibitive to perform the FDR for all *p*-values of the genome-wide analysis, so we only used *p*-values where the SNP–gene distance was less than 1 Mb *(FDR-cis)* to interrogate the *cis* signal. We identified 40 genes that had at least one SNP with *q* < 0.05. Of these, 38 were overlapping with the 63 genes that were individually called significant in the permutation analysis *(Perm-cis-enrich).* The dependence of SNPs due to linkage disequilibrium may be considered a problem, but as described in Remark D in [24], this dependence may actually lead to conservative estimates of significant genes. However, to account for the effects of dependence of SNPs in associated regions, and be even more conservative, we removed from the FDR analysis all *p*-values of SNP–gene tests within 100 kb from the lowest *p-*value for each of the 40 genes above *(FDR-cis-trim).* When new FDR thresholds were assigned, only 20 genes remained significant, although the remaining 20 were very close to the new threshold. All 20 genes were in the pool of 63 genes identified by the *Perm-cis-enrich* method. The reason that only 20 genes remained significant is because although we removed the dependence in regions that had a strong signal, we did not remove the dependence in regions with null signal and these regions are more common. So although only 20 genes were left after the *FDR-cis-trim* analysis, the 40 genes detected with the original *FDR-cis* analysis should be considered as a set of genes with significant *cis* signal.

### Overlap between Multiple-Test Correction Methods

Table 1 summarizes the numbers of genes identified by each method and the overlaps of associated genes. The biggest discordance was that between *Bonf-gw* and *Perm-gw*. *Bonf-gw* identified three *trans* associations that were not considered significant by *Perm-gw*. This is due to the presence at each of these three expression phenotypes of one or two individuals with phenotypic outliers (very slightly exceeding the mean + three standard deviations) that caused extreme *p-*values with a few SNPs. The presence of these phenotypic outliers violates the normality assumptions of the linear regression model, thus the *p*-values obtained are not valid. Such problems are not accounted for by the Bonferroni correction if phenotypic distributions are not filtered in advance but can be accounted for by permutation-based thresholds. All analyses in *cis* showed that ten associated genes were considered significant across all methods that focused on the *cis* signal only *(Perm-cis, Perm-cis-enrich, FDR-cis, FDR-cis-trim).* Two of these ten genes, CPNE1 and CSTB, were also shown to have significant *cis* signals in a previous study [10,12], corroborating the validity of the observed signals. Interestingly, the *FDR-cis* and *Perm-cis-enrich* analyses showed that it is likely that more than ten genes have a significant *cis* signal. The overlap of these methods was almost 100% (38 out of 40 genes identified in the *FDR-cis* analysis were included in the 63 genes significant by *Perm-cis-enrich*). It is worth noting that the *FDR-cis* analysis with *q* = 0.05 predicts that 95% of the 40 genes (i.e., 38 genes) will be real signals, which is exactly the number that overlaps with *Perm-cis-enrich*. Overall, we observed a very good concordance between the different statistical methods that were used to assign significance based on multiple test correction.

**Table 1:**
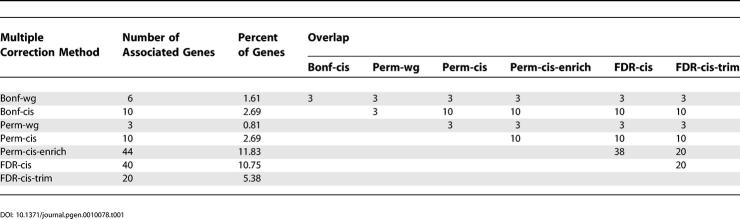
Comparison of Multiple-Test Correction Methods

### Power and the Magnitude of Effects

The small sample size and the large number of SNPs and phenotypes bring challenges to the interpretation of the significant effects we detect. Our rationale to focus on *cis* effect stems from the fact that *cis* regions are candidate regions to contain regulatory elements that influence the proximal genes, while it is harder to select a priori such regions from the rest of the genome. Therefore, our ability to detect more *cis* effects is not necessarily because larger effects occur more frequently in *cis* regions, but because we had more power when performing the analysis in *cis* regions. In order to estimate the power given the experimental design, one can perform power calculations. However, such calculations require many assumptions about the data. An alternative, given the multiple test correction we have performed, is to describe the magnitude of the effect that each of our analyses can detect. The adjusted r^2^ values from the linear regression provide an estimate of the fraction of variation that is explained by an individual SNP. Based on the adjusted r^2^ values from the linear regression performed for the SNPs with the lowest *p-*values *(cis* or *trans),* we have more than 80% power to detect effects of r^2^ ≥ 0.50 when genome-wide correction *(Bonf-gw* or *Perm-gw)* is applied and the type I error is set to 0.05. When we perform the correction based on the 1-Mb distance from the corresponding gene, we have more than 80% power to detect effects with r^2^ ≥ 0.22. The magnitude of individual effects of SNPs for each gene is illustrated in Table 2. The average r^2^ value for the three genes deemed significant by *Perm-gw* was 0.58; average r^2^ was 0.48 for the ten genes called significant by the *Perm-cis* analysis, and average r^2^ was 0.27 for all 63 genes that had a significant *cis-* signal according to at least one of the methods. It should be noted that estimates of effect size are often biased upwards in QTL studies [27]. Overall, our analysis can only detect large effects, and larger sample sizes are needed for the detection of smaller but not necessarily less important regulatory effects.

**Table 2:**
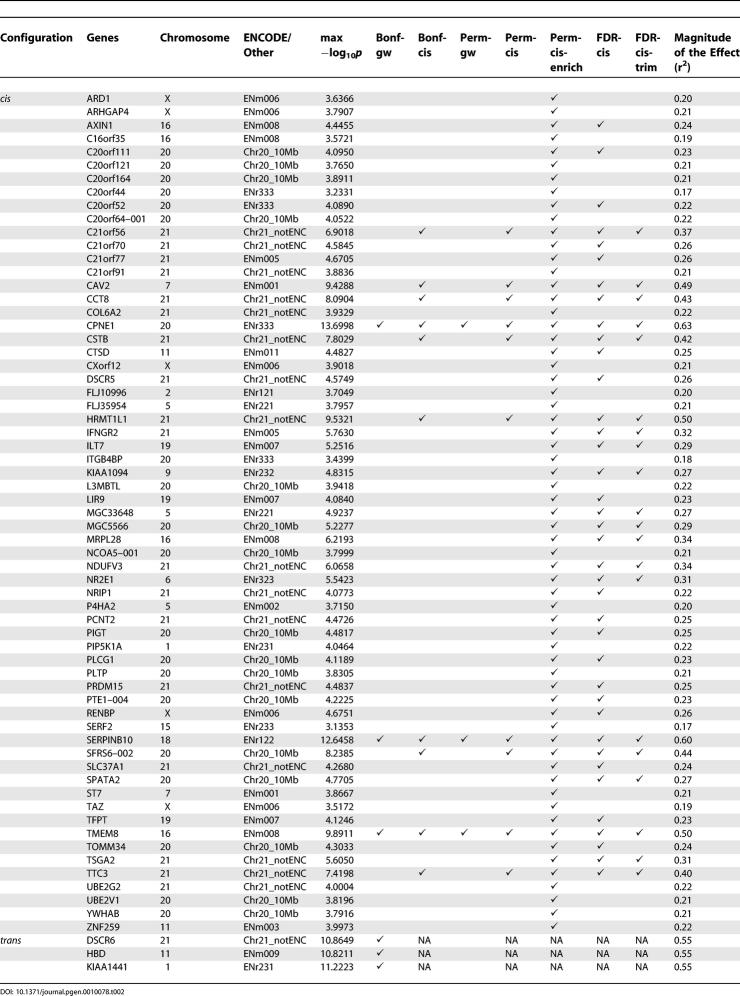
Genes with Significant *cis* and *trans* Associations

### Specific Examples of Associations

The most highly significant association signal detected was with SNPs *cis-* to the *SERPINB10* gene, located in a region of Chromosome 18 that harbors five additional members of the serine (or cysteine) proteinase inhibitor, clade B (ovalbumin) gene family (Figure 3A). The additive model identified SNPs with highly significant association with transcript levels of *SERPINB10* distributed across two LD blocks; furthermore both probes for this gene exhibited the same significant signal (unpublished data). The majority of significantly associated SNPs are located immediately upstream of the transcription start site, while others are in the coding locus itself and immediately downstream (Figure 3A). Linkage disequilibrium is strong in this region (Figure 3A) and thus the stretch of SNPs exhibiting a similar highly significant association may actually be generated by only one or a few variants that cause the difference in expression. The fact that there are significantly associated *cis-* SNPs in two regions of high LD surrounding this locus, suggests the possibility of two independent regulatory variants.

**Figure 3:**
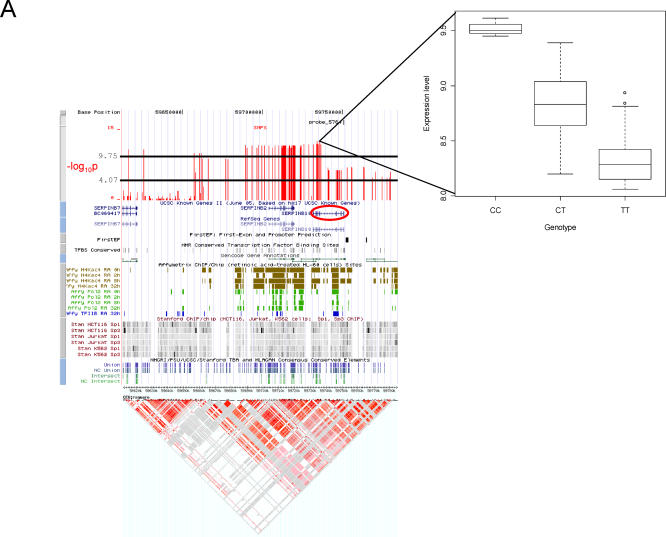
Examples of *cis-* Associations from the Genome-Wide and High-Density SNP Maps (A) Genomic location of associated SNPs close to the *SERPINB10* gene. Custom tracks in the UCSC genome browser (http://genome.ucsc.edu) show the location of the Illumina probe and proximal SNPs in the context of genome annotation. The lower horizontal black line indicates the −log_10_ *p* threshold where the corresponding *q-*value is 0.05 (i.e., any SNPs with values −log_10_ *p* that meet or exceed this threshold are significant at the *q* = 0.05 level), and the upper line is the Bonferroni genome-wide threshold. Additional tracks describe known genes, first-exon and promoter predictions, conserved transcription factor binding sites, Gencode genes, RNA polymerase 2, and Transcription factor 2 binding sites, identified by Affymetrix ChIP/chip experiments, and Sp1 and Sp3 binding sites identified by Stanford's ChIP/chip experiments. Consensus conserved elements are shown in the final track. HapMap LD information below is for the CEU individuals and suggests that there are two conserved haplotype clusters in this region.

**Figure 3:**
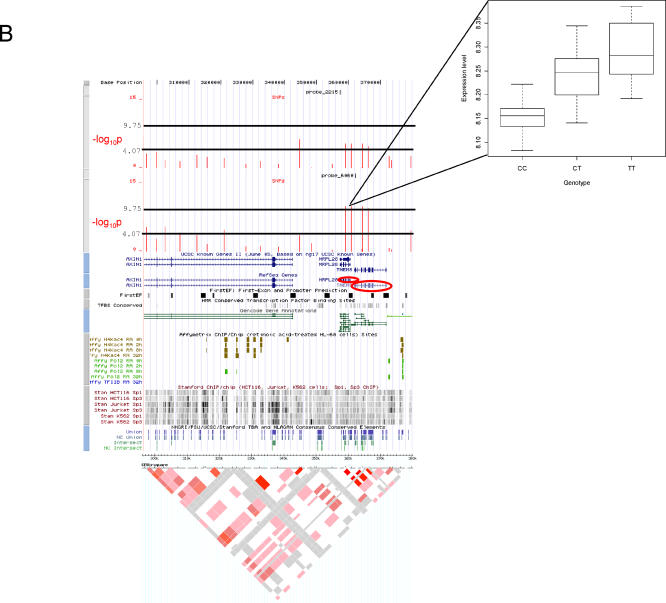
Continued (B) Genomic location of associated SNPs close to the *TMEM8* and *MRPL28* genes. Note the correlation between the *p*-values for the two genes. Custom tracks in the UCSC genome browser show the location of the Illumina probe and proximal SNPs in the context of genome annotation. The lower horizontal black line indicates the −log_10_ *p* threshold where the corresponding *q-*value is 0.05 (i.e., any SNPs with values −log_10_ *p* that meet or exceed this threshold are significant at the *q* = 0.05 level), and the upper line is the Bonferroni genome-wide threshold. Additional tracks describe known genes, first-exon and promoter predictions, conserved transcription factor binding sites, Gencode genes, RNA polymerase 2, and Transcription factor 2 binding sites, identified by Affymetrix ChIP/chip experiments, and Sp1 and Sp3 binding sites identified by Stanford's ChIP/chip experiments. Consensus conserved elements are shown in the final track. HapMap LD information below is for the CEU individuals (http://genome.ucsc.edu) [39].

One gene that is consistently significant in genome-wide corrections is TMEM8. The gene is found on Chromosome 16 and exhibits significant *p-*values with the highest being 1.3 × 10^−10^ for SNPs very close to the coding sequence. An intriguing observation is that another gene immediately 3′ to TMEM8, MRPL28 is also associated with the same SNPs but with higher but still significant *p*-values (6.0 × 10^−7^) (Figure 3B). TMEM8 encodes a transmembrane protein whereas MRPL28 encodes a ribosomal protein and is a gene transposed from the mitochondrial genome, so there is no apparent biological relationship between them. Both genes are in the same orientation so an intriguing scenario is that there are transcripts spanning both genes that are driven from a single promoter, and this is the reason for such high correlation in expression. Such phenomena are now commonly observed in the human and other genomes [28]. However, expressed sequence tag data do not support this idea since none of them show a transcript that spans both genes. Another exciting scenario, from an evolutionary point of view, is that MRPL28 has “landed” at some point in time in the vicinity of TMEM8 and recruited already existing regulatory regions in the region, one of which was a TMEM8 regulatory region. Subsequent segregating variation in this regulator may have then affected the expression of both genes.

### SNPs in Hybridization Probes

For each of the 40 genes showing at least one significant association according to the *FDR-cis* analysis, we considered the possibility of the influence on expression levels of SNPs located at the probe binding sequence. We searched the sequences of all probes for known sequence variants and examined the entry in dbSNP (version 125; http://www.ncbi.nlm.nih.gov/projects/SNP) to assess the likelihood of the variant being a real SNP. We identified a total of 18 variants in sequences of probes for 12 of the 40 genes; three of these genes are in the set of ten loci that exhibited significant *cis-* signal according to all *cis* methods. Of these 18 variants, eight had no confidence that they were actual SNPs (i.e., there is no frequency information for any population). Two SNPs had no frequency information, but were identified twice by two different centers. Eight variants are SNPs with genotype frequencies, only three of which have been genotyped in the HapMap CEU population. Of the three SNPs genotyped in the HapMap project, one is monomorphic in CEU and the other two are in high LD with the SNPs exhibiting the strongest significant association in *cis* for the genes AXIN1 and HRMT1L1, suggesting that the observed *cis* associations for these two genes may be due to the SNP presence in the probe sequence. Other authors have noted that even with 60-mer probes, a single SNP underlying the probe sequence can lead to spurious *cis*- associations [29]. Although this is a technical artifact, it illustrates that the experimental and statistical methodologies described here are able to detect signals of differential hybridization with one nucleotide mismatch in the 50-mer probes.

## Discussion

Our analysis suggests that there is an abundance of common genetic variation that explains gene expression differences among individuals. From a total of 374 genes with above-background signal interrogated in 60 individuals, we can detect genetic variants with significant effects on transcript level for ten to 40 genes in *cis*. *Trans* signals in three genes are only supported by one of the two statistical methods employed for genome-wide analysis (Bonferroni and permutations) and are more likely to be false positives. We therefore observed that the majority of detectable signals are caused by SNPs located *cis-* to the gene, and the signal is consistent with an additive effect of the causal variant. This observation is consistent with previously published studies that found a significant proportion of the intraspecific differences in transcript level are located *cis-* to the gene in question [8,10]. The fact that only a small fraction of the genes had significant signal is likely a result of reduced power due to the small sample size. Moreover, testing additional cell types is likely to reveal more regulatory variants. In addition, when we define significance thresholds by focusing on proximal SNPs we detect many more significant associations. This suggests that if we apply differential weighting of SNPs based on proximity to the gene we may be able to enhance our ability to detect significant *cis* effects [30]. These models are still in an exploratory stage. An intriguing possibility is that some of these significant regions indicate the presence of an additional copy of the gene *(cis* or *trans)* due to copy number polymorphisms [CNPs; 31,32], and in fact we have identified the haplotype with the additional copy of the gene. For example, C16orf15 which is significant only for *Perm-cis-enrich* overlaps with a known copy number variant (http://projects.tcag.ca/variation). Our methodology may thus help identify the map position of excess gene copies. Such cases will be examined when we have copy number polymorphism data for these individuals. Other alternative explanations are that the mapped SNPs tag variants that affect mRNA stability, mRNA trafficking, and other post-transcriptional effects.

In order to assess alternative approaches to dealing with the massive multiple testing problem faced here, we have applied three standard methodologies. The fact that there is substantial overlap of the signals detected from all three suggests that most signals in our analysis are robust and more likely to be true positives, given the distinct theoretical basis for the Bonferroni, permutation, and FDR tests. Such a test of robustness to assumptions in multiple test correction may ultimately be necessary when the number of tests is so large. This will become an even bigger problem when one accounts for potential interactions between SNPs, something we have not addressed in the present study. Given the fact that each phenotype has its own properties of variance and inheritance, it seems unlikely that genome- and experiment-wide thresholds provide the optimal means for assessing significance. Experimental validation of such targets is the next priority [29], and in order for it to be informative one has to consider not only the top few signals but sample from a wide range of significance levels. In fact, it is not yet obvious that what appears to be the most statistically significant effect is the most biologically relevant effect. True validation will require the elucidation of the complete biological effect, rather than a first-pass, low sensitivity, experimental validation procedure.

Perhaps the most promising aspect of studies of gene expression variation mapping is the impact it can have on interpretation of functional genomic information and functional variation. For instance, most of the experimental methodologies that identify regulatory regions in humans rely on experimental procedures that, although they can elucidate the regulatory potential of a region (e.g., binding assays, transfection assays, etc.), they are not able to pinpoint the gene which interacts with the candidate regulatory region. If one couples these experimental procedures with the methodology described in the present study, direct links between regulatory regions and genes can be identified, which will facilitate biological interpretations. In addition, such an approach can greatly facilitate the interpretation of genotype-phenotype (disease) associations when the mapped regions fall into non-coding regions of the genome with no annotated functional DNA.

We have demonstrated that it is feasible to map genetic variants affecting gene expression by genome-wide association testing. The HapMap samples, which have been genotyped for more than 3.5 million SNPs, offer a powerful resource for such gene expression studies as well as for other studies that measure quantitative traits at the cellular level. It has been hypothesized that the genetic basis of complex traits and diseases is largely regulatory [33,34] and such studies may point to potential disease variants. The sets of genes screened in this study are of a strategic nature. The ENCODE regions will soon have accumulated large amounts of functional data, some of which are relevant to gene regulation [20]. We already see patterns of correlation between the location of the associated SNPs and the regulatory active regions (see Figure 3A), which will greatly enhance our ability to interpret the effect of associated variants. Also, Trisomy of HSA21 causes Down syndrome (i.e., gene dosage effect) and the elucidation of expression allelic variants will facilitate the discovery of genes associated with the variable Down syndrome phenotypes such as the congenital heart defect present in 40% of patients with Down syndrome [35]) The 10 Mb region of chr20 is known to be associated with type II diabetes and obesity [36], and the discovery of allelic expression variants may reveal potential causal candidates for this association. Mapping of gene expression phenotypes in cell lines of heavily genotyped samples will provide a baseline resource that will greatly facilitate the fine mapping of disease variants in human populations [37]. We have demonstrated that the use of multiple methods for statistical inference greatly facilitates the interpretation of results and generates stronger candidates for experimental follow-up. Our results have important implications, both for the regulatory landscape of the human genome, as well as for the choice of the type of variation one needs to interrogate for disease association studies.

## Materials and Methods

### RNA preparation.

Total RNA was extracted from the 60 HapMap parental CEU lymphoblastoid cell lines (Coriell, Camden, New Jersey, United States). Two one-quarter scale Message Amp II reactions (Ambion, Austin, Texas, United States) were performed for each RNA extraction using 200 ng of total RNA. Biotin-16-UTP (Perkin Elmer, Wellesley, California, United States) made up half of the UTP used in the in vitro transcription (IVT) reaction. cRNA yields were quantified using RiboGreen (Invitrogen, Carlsbad, California, United States). 1 μg of the cRNA was then hybridized to an array.

### Gene expression quantification.

We designed a custom expression array bead pool with 1,433 unique bead types (two for each of 630 genes, plus controls) each with ~50,000 50-mer probes using Illumina Bead Arrays (Illumina, San Diego, California, United States). Our custom arrays include 321 protein coding ENCODE genes (excluding Chromosome 21), 191 protein coding genes from Chromosome 21, and 118 protein coding gene transcripts from 20q12–13.2. Bead pools were added to bundles of 50,000 fiber optic filaments which were arrayed into an 8×12 Sentrix Array Matrix (SAM) so that 96 arrays could be run in parallel. Each bead type (probe) is present on a single array on average 30 times. Each of the two IVT reactions from the 60 samples was hybridized to three arrays, so that each cell line had six replicate hybridizations. Twelve cell lines had two extra replicates run from one of their IVT reactions, giving them eight replicates, for a total of 384 arrays. cRNA was hybridized to arrays for 18 h at 55 °C before being labelled with Cy3-streptavidin (Amersham Biosciences, Little Chalfont, United Kingdom) and scanned with a Bead Station (Illumina).

### Post-experimental raw data processing. Normalization.

With the Illumina bead technology, a single hybridization of RNA from one cell line to an array produces on average approximately 30 intensity values for each of 1,433 bead types. These background-corrected values for a single bead type are subsequently summarized by the Illumina software and output to the user as a set of 1,433 intensity values for each individual hybridization. These raw data were then normalized on a log scale using a quantile normalization method [38]. The expression measurements from the 384 array bundles then have the same distribution.

### Averaging replicates.

In our experiment, each cell line was hybridized to six or eight arrays, thus resulting in six or eight reported intensity values (as averages of the values from the 30 beads per probe) for each of the 1,433 bead types. To combine data from our multiple replicate hybridizations, we averaged these normalized intensity values for each bead type to obtain a single value for each of the 1,433 bead types for each individual. These averages (for each probe, across replicates for each individual) of normalized average values are the values used in subsequent analyses.

### Association analyses.

The whole genome association analysis employed 753,712 SNP genotypes with minor allele frequency above 5% from the HapMap 5k map (version 16b). Of the 1,405 probes (excluding control probes), we chose the 688 most variable probes (corresponding to 374 unique genes) to use in the association analyses after excluding probes with extreme outliers.

For each of the selected probes and for each SNP, we fitted the following model: the genotype *X_i_* of individual *i* at the given SNP may be classified as one of three states: *X_i_* = 0, 1, or 2 for homozygous rare, heterozygous and homozygous common alleles, respectively. For this additive model, we fitted a linear regression of the form

*Y_i_* = *b_0_* + *b_1_X_i_* + *ɛ_i_*

where *Y_i_* is the normalized log-expression level of the probe for individual *i, i* = 1,…, 60, and *ɛ_i_* are independent normally distributed random variables with mean 0 and constant variance. We report the nominal, parametric *p-*value of the test of no association, i.e., *b_1_ = 0.*

### Multiple-test correction.

We have employed three approaches for multiple-test correction, namely Bonferroni, assignment of significance based on permutation of phenotypes to the genotypes, and FDR. The first two were applied to both genome-wide analysis and *cis* analysis (1 Mb) and the FDR was applied only to *cis* analysis (1 Mb) due to the computational limitations, since the number of genome-wide *p-*values was too large to be analyzed.

## Supporting Information

Figure S1 Chromosomal Location of 63 Genes Chromosome banding, karyotype cartoon showing the location of the 321 ENCODE genes, the 191 Chromosome 21 genes, and the 118 genes from 20q12–13.2.
(35 KB PPT)

Figure S2 Correlation between Gene Expression Phenotype Measurements as Quantified by Pairs of Probes Corresponding to the Same Gene Included are pairs of probes from the set of 688 analyzed probes.
(45 KB PPT)

Figure S3 Examples of *cis-* Associations from the Genome-Wide and High-Density SNP Maps; CPNE1 Locus (127 KB PDF)

### Accession Numbers

The expression data reported in this paper have been deposited in the Gene Expression Omnibus (GEO) (http://www.ncbi.nlm.nih.gov/geo) database (Series Accession Number GSE3612). The accession numbers of the genes mentioned herein include: AXIN1 (NM_181050.1), CPNE1 (NM_152926.1), CSTB (NM_000100.2), HRMT1L1 (NM_001535.1), MRPL28 (NM_006428.2), *SERPINB10* (NM_005024.1), and TMEM8 (NM_021259.1).

## Abbreviations

CEU: Utah residents with ancestry from Northern and Western Europe
ChIP: chromatin immunoprecipitation
FDR: false discovery rate
LD: linkage disequilibrium
SNP: single nucleotide polymorphism
