# Supplementary material for: Genome-Wide Associations of Gene Expression Variation in Humans
Source: PLoS Genet. 2005 Dec 16;1(6):e78. doi: 10.1371/journal.pgen.0010078 (PMC1315281; doi:10.1371/journal.pgen.0010078)
Supplement: Supplementary file 1 [file pgen.0010078.sg003.pdf]

Figure S3.

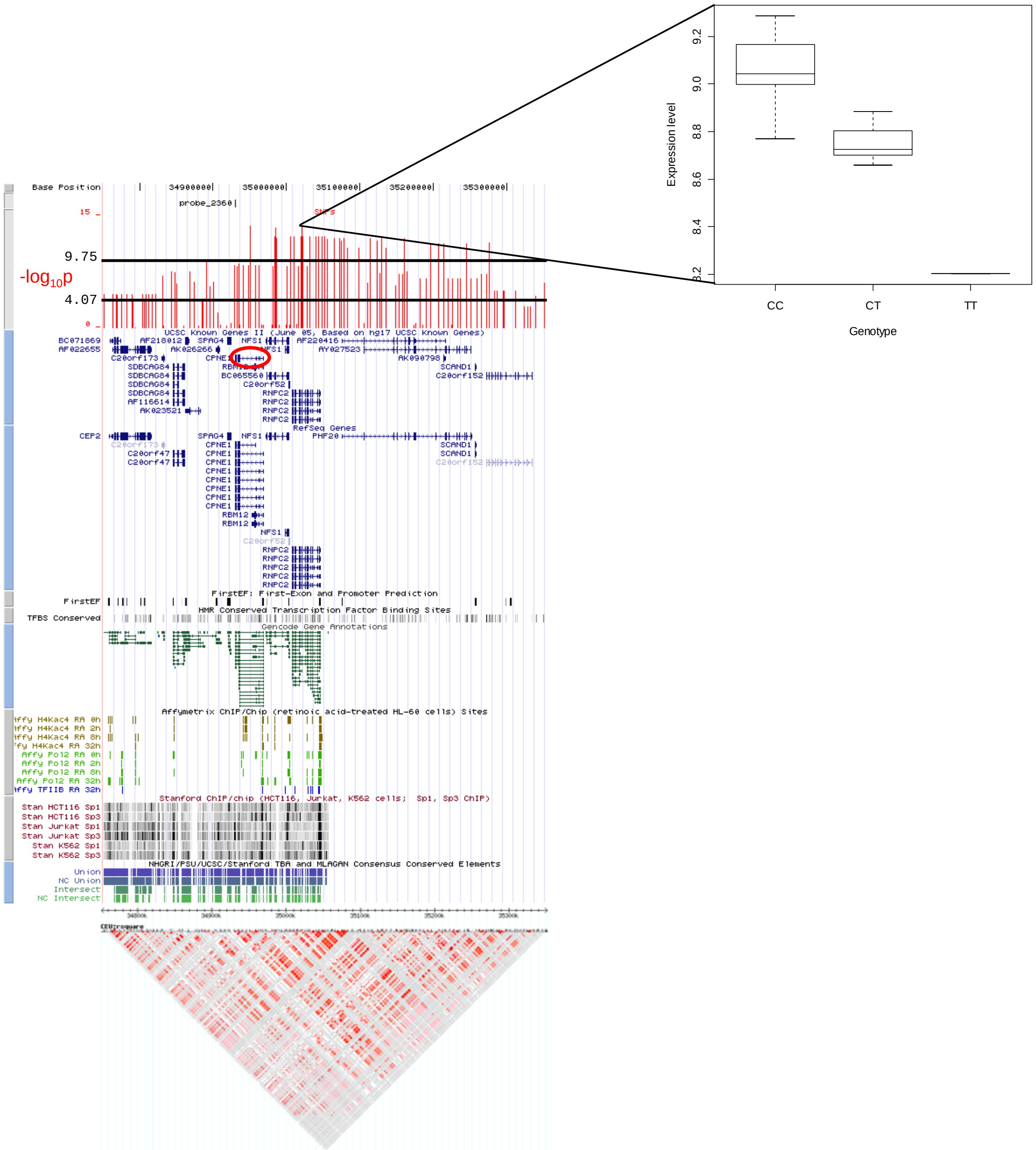

9.75
-log10p
4.07
Expression level
8.2
8.4
8.6
8.8
9.0
9.2
CC
CT
TT
Genotype
